# Overexpression of Rab11-FIP2 in colorectal cancer cells promotes tumor migration and angiogenesis through increasing secretion of PAI-1

**DOI:** 10.1186/s12935-018-0532-0

**Published:** 2018-03-09

**Authors:** Wenjie Dong, Xinai Wu

**Affiliations:** Department of Medical Oncology, the First Affiliated Hospital, Zhengzhou University, 1 East Jianshe Road, Zhengzhou, 450052 Henan People’s Republic of China

**Keywords:** Rab11-FIP2, Colorectal cancer, Migration, PAI-1

## Abstract

**Background:**

Rab11 family-interacting protein 2 (Rab11-FIP2) can interact with MYO5B and plays an important role in regulating plasma membrane recycling. However, little is known about the clinical significance of DUSP2 in colorectal cancer (CRC).

**Methods:**

In this study, we investigated Rab11-FIP2 expression by immunohistochemistry in 125 patients with colorectal cancer. Conditioned media containing all secreted factors was harvested. Chemokine secretion and expression were analyzed by Chemi-array.

**Results:**

We found that the expression level of Rab11-FIP2 was significantly increased in colorectal cancer tissues and high expression of Rab11-FIP2 was closely correlated with nodal metastasis in colorectal cancer patients. Rab11-FIP2 overexpression promoted colorectal cancer metastasis in vitro and in vivo. Finally, we demonstrated that Rab11-FIP2 overexpression may contribute to increased secretion of PAI-1 in human colorectal cancer cells.

**Conclusions:**

Our findings reveal a novel mechanism underlying the role of Rab11-FIP2 in colorectal cancer dissemination, suggesting that targeting Rab11-FIP2 might be a promising therapeutic strategy for CRC.

**Electronic supplementary material:**

The online version of this article (10.1186/s12935-018-0532-0) contains supplementary material, which is available to authorized users.

## Background

Rab11-FIP2 is a member of a family of Rab11-binding proteins (Rab11-FIPs) that have been implicated in the function of plasma membrane recycling [1]. The interaction of Rab11-FIP2 with both Rab11a and MYO5B regulates recycling endosome trafficking [2, 3]. Growing evidence demonstrates that Rab11-FIP2 plays a substantial regulatory role in tumor progression and metastasis. For example, Rab11-FIP2 promotes colorectal cancer migration and invasion by regulating PI3K/AKT/MMP7 signaling pathway [4]. Our previous study also demonstrated that Rab11-FIP2 was significantly increased in gastric cancer tissues [5]. Rab11-FIP2 overexpression promoted epithelial–mesenchymal transition (EMT) in a manner associated with gastric cancer metastasis in vitro and in vivo. These results suggested that Rab11-FIP2 had important roles in tumor progression and metastasis.

It was reported that Rab11-FIP2 regulated CXCR2 recycling and receptor-mediated chemotaxis and that passage of internalized CXCR2 through Rab11a-positive recycling system is critical for physiological response to a chemokine, suggesting that Rab11-FIP2 may play a role in chemotaxis [6]. PAI-1 and IL-8 were identified to be correlated with tumor grade/stage and prognosis [7–9]. PAI-1 and IL-8 were also identified to be correlated with tumor angiogenesis [10]. In this study, we showed that Rab11-FIP2 overexpression may contribute to increased secretion of PAI-1 in human colorectal cancer cells. Furthermore, pharmacological inhibition of PAI-1 resulted in the reduction of cell migration and colony formation, and the induction of apoptosis in CRC cells overexpressing Rab11-FIP2.

## Materials and methods

### Antibody array detection

In total, cells were plated into 10-cm culture dishes for 24 h. The media were replaced with serum deprived media containing 0.1% fetal bovine serum, and the cells were cultured for 48 h. The cell lysate and culture supernatants were collected. Samples were labeled with biotin and incubated with Human cytokine Antibody Arrays (ARY005B, R&D Systems, USA). Representative images from two independent experiments were shown. Protein concentration of each sample was determined using the BCA kit (Pierce) per manufacturer’s instructions.

### Patients, tissue microarray, immunohistochemistry and cell culture

Tissue microarray of 125 colorectal cancer tissues with clinical outcome was purchased from Superchip (Shanghai, China). Non-tumor samples from the macroscopic tumor margin were isolated at the same time and used as the matched adjacent non-neoplastic tissues (> 5 cm). All samples were obtained their informed consent and with institutional review board approval of the hospital. All patients obtained a confirmed diagnosis of colorectal carcinoma after resection.

The primary antibodies used were antibodies against Rab11-FIP2 (1:250 dilution; Abcam). Negative controls were treated identically but with the primary antibody omitted. We set each score as follows: negative, score 0; weak, score 1; moderate, score 2; strong, score 3. We classified these samples into 2 groups: 0–1, negative (−); 2–3 positive (+). HCT15 and HCT116 cell lines were obtained from American Type Culture Collection. HCT15 and HCT116 cells were cultured in a normoxia (37 °C and 5% CO_2_) condition. All media were supplemented with 10% FBS and antibiotics (penicillin, streptomycin, and gentamycin) unless otherwise indicated. Human umbilical vein endothelial cells (HUVEC) were cultured under company-recommended conditions.

### RT-PCR and DNA constructs

Total cellular RNA was prepared by using TRIzol reagent (Invitrogen) and RT-PCR was performed. The following PCR primers were used: PAI-1 forward primer: reverse primer: 5′- AGCTCCTTGTACAGATGCCG-3′; 5′-ACAACAGGAGGAGAAACCCA-3′. GAPDH forward primer: 5′-AACTTTGGCATTGTGGAAGG-3′; reverse primer, 5′-ACACATTGGGG GTAGGAACA-3′ [11]. PCR products were electrophoresed on 1% agarose gels and visualized by ethidium bromide staining under UV trans-illumination. cDNA was made with oligo-dTTP using M-MLV reverse transcriptase. PCR amplification was performed under optimized conditions for each primer pair. The full length of Rab11-FIP2 was amplified (forward primer: 5′-ATGATGCTGTCCGAGCAAGCC-3′; reverse primer: 5′-TTAACTGTT AGAG AATTTGCCAGC-3′) and then cloned into the pCMV-4 vector. The correctness of the construct was confirmed by sequencing. An empty vector was used as a negative control. The transfected cells were selected under 800 mg/mL G418 (Sigma) for 3–5 weeks. Stably transfected clones were validated by RT-PCR and Western blot analysis.

### Wound-healing assays

Cells were seeded in six-well plates and incubated until 90% confluence in serum-free medium before wounding. A 200-μL tip was used to make a vertical wound, and the cells were then washed three times with PBS to remove cell debris. Cell migration into the wounded area was monitored by microscopy at the designated times.

### Anchorage-independent growth and Transwell migration assays

For anchorage-independent growth assay, 2 × 10^4^ cells were plated in 0.3% low melting point agar/growth medium onto 6 cm dishes with a 0.6% agar underlay. After 4 weeks, the number of colonies was determined. For transwell migration assays, HCT15 and HCT116 5 × 10^4^ cells in serum-free medium were added to the upper chamber of each insert (BD Biosciences). In this assays, medium supplemented with serum was used in the lower chamber. After incubation in a normoxia (37 °C and 5% CO_2_) chamber for 24 or 48 h, the cells on the upper surface were removed, and the cells on the lower surface of the membrane were fixed in 100% methanol for 15 min, air dried, stained with 0.1% crystal violet, and counted under a microscope to calculate relative numbers. Nine random fields were analyzed per insert. Each experiment was conducted in triplicate in three independent experiments.

### Electron microscopy and scanning electron microscopy

For scanning electron microscopy analysis, cells were washed with PBS and fixed with 2.5% glutaraldehyde for 1 h at room temperature. Samples were dehydrated in graded ethanol solutions from 50 to 100% and in hexamethyldisilazane (HMDS, Ted Pella, Redding, CA, USA), then sputter-coated with platinum. Scanning electron microscopy (SEM) was performed on an Analytic FEI Quanta FEG 200 microscope (FEI, Hillsboro, OR, USA) with an acceleration voltage of 15 kV in a pressure of 1 × 10^−5^ Torr.

### Western blotting

Samples (20 µg) of the cell lysate were subjected to 10% SDS-PAGE gel electrophoresis, after which the resolved proteins were transferred to nitrocellulose membranes (Amersham Biosciences). The membranes were then blocked with 5% non-fat milk and 0.1% Tween 20 in Tris-buffered saline, followed by incubation with primary antibody (1:1000 dilution) at 4 °C overnight. The membrane was washed thoroughly and then incubated with HRP-conjugated secondary antibody (1:10,000 dilution; Sigma) for 2 h at room temperature. The protein was visualized by chemiluminescence (Pierce).

### Evaluation of apoptosis

Apoptosis was detected by flow cytometric analysis of Annexin V staining. Annexin V–FITC versus PI assay was performed as previously reported. Briefly, adherent cells were harvested and suspended in the Annexin-binding buffer (1 × 10^6^ cells/mL). Thereafter, cells were incubated with Annexin V–FITC and PI for 15 min at room temperature in the dark and immediately analyzed by flow-cytometry. The data are presented as bi-parametric dot plots showing Annexin V–FITC green fluorescence versus PI red fluorescence.

### Evaluation of lung metastases of CRC cells injected intravenously

Nude mice (4–5 weeks old) were maintained in SPF laboratory Animal Central. All animals’ maintenance and procedures were carried in accordance with the institute’s guidance on animal experimentation. 15/FIP2 and 116/FIP2 cells (1 × 10^6^) were injected into the tail veins of mice. Eight weeks later, the mice were killed, the lung tissues were fixed, paraffin embedded and 5 μm tissue sections were stained with hematoxylin and eosin (H&E). The number of macroscopic and microscopic metastatic nodules in the lungs was counted.

### Tube formation

HUVECs (2 × 10^4^) were plated onto matrigel-coated (10 mg/mL, BD Pharmingen) 12-well plates with condition media of 116/FIP2 cells. After 12 h of the incubation at 37 °C, HUVECs were fixed with 4% paraformaldehyde and the formation of capillary-like structures was captured under a light microscope (OLYMPUS, Japan). The number of branch points of the tube structures, as the degree of angiogenesis, was counted in three fields at 100× magnification. PAI-1 neutralizing antibody (R&D System, 10 µg/mL) was used for inhibition of PAI-1 in this analysis.

### Statistical analysis

Pearson Chi-Square test and one-ANOVA were used for statistical analysis of group differences. *P* values less than 0.05 was considered significant.

## Results

### Rab11-FIP2 expression is increased in colorectal cancer and is associated with nodal metastasis

**Fig. 1:**
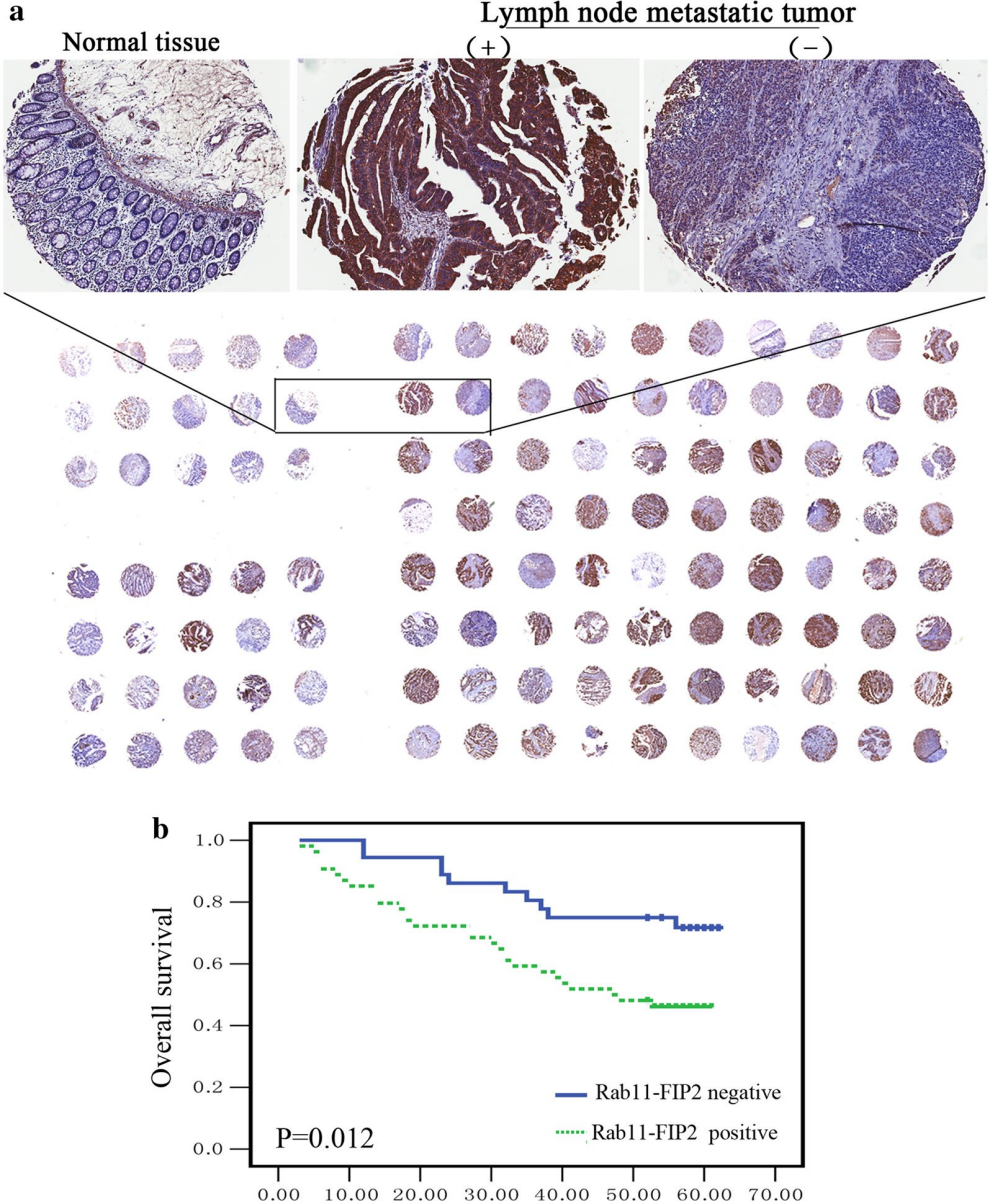
Association between Rab11-FIP2 expression and tumor lymph node metastasis in colorectal cancer patients. **a** IHC analysis shows strong Rab11-FIP2 expression in colorectal cancer with lymph node metastasis, whereas colorectal cancer without lymph node metastasis and adjacent normal epithelia show weak Rab11-FIP2 expression. **b** Kaplan–Meier survival curve of colorectal cancer patients with low and high Rab11-FIP2 expression

To determine Rab11-FIP2 expression in colorectal cancer, we analyzed a tissue microarray containing primary colorectal cancer and paired adjacent normal tissue. Overall, immunohistochemical (IHC) analysis revealed that Rab11-FIP2 levels are significantly more elevated in colorectal cancer, as compared with their normal epithelial tissue (Fig. 1a). The data on the IHC are summarized in Table 1. There was a significant correlation between elevated expression of Rab11-FIP2 and nodal metastasis (*p* < 0.001). We also found that Rab11-FIP2 was upregulated in CRC with advanced clinical stage (III + IV) (*p* = 0.026). We also analyzed the Rab11-FIP2 expression according to tumor size, cancer differentiation levels and age. However, none of the parameters was significantly different among their subgroups. Furthermore, we also analyzed the effect of Rab11-FIP2 expression on cancer-related survival in colorectal carcinoma. The log rank test demonstrated that tumors with the high Rab11-FIP2 expression were associated with short overall patient survival, whereas patients with tumors displaying low level of Rab11-FIP2 expression showed a better clinical outcome (Fig. 1b).

**Table 1:** Clinical characteristics of colorectal cancer patients according to expression status of Rab11-FIP2

Group	Rab11-FIP2 expression		*p* value
	Positive	Negative	
Cancer tissues	79	46	
Gender			
Male	32	23	0.454
Female	47	23	
Age (year)			
< 60	40	25	0.542
≥ 60	39	21	
Differentiation			
Well	10	3	0.293
Moderate	36	23	
Poor	33	20	
Lymph-node metastasis			
Yes	53	6	< 0.001
No	26	40	
Size (cm)			
< 4	41	23	0.785
≥ 4	38	23	
Stage			
I + II	36	30	0.026
III + IV	43	16	

### Rab11-FIP2 promotes migratory capacities of colorectal cancer cells in vitro and in vivo

**Fig. 2:**
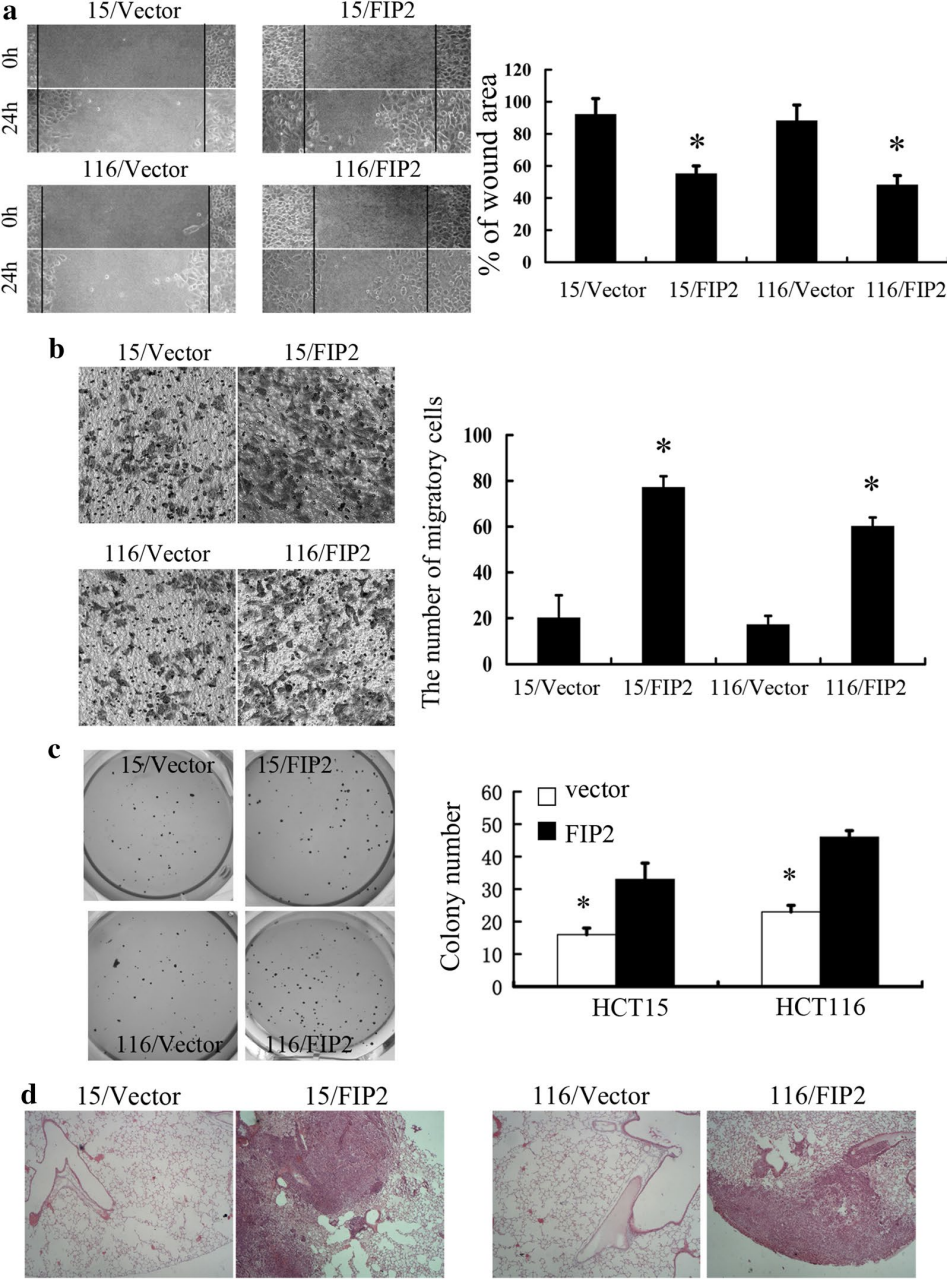
Rab11-FIP2 is important for motility and metastasis of HCT15 and HCT116 cells. **a** Overexpression of Rab11-FIP2 affects HCT15 and HCT116 cells migration in an in vitro wound assay. Average migratory width of three independent experiments is shown (*p < 0.05). **b** 15/FIP2 and 116/FIP2 cells or control vector cells were subjected to Transwell migration assays. The migrated cells through the membrane of each cell line are quantified. All results are from three independent experiments (*p < 0.05). **c** Overexpression of Rab11-FIP2 promoted colony formation in CRC cells. **d** Pathology analyses showed that overexpression of Rab11-FIP2 significantly increased lung metastasis of HCT15 and HCT116 cells in nude mice lung seeding assay

**Fig. 3:**
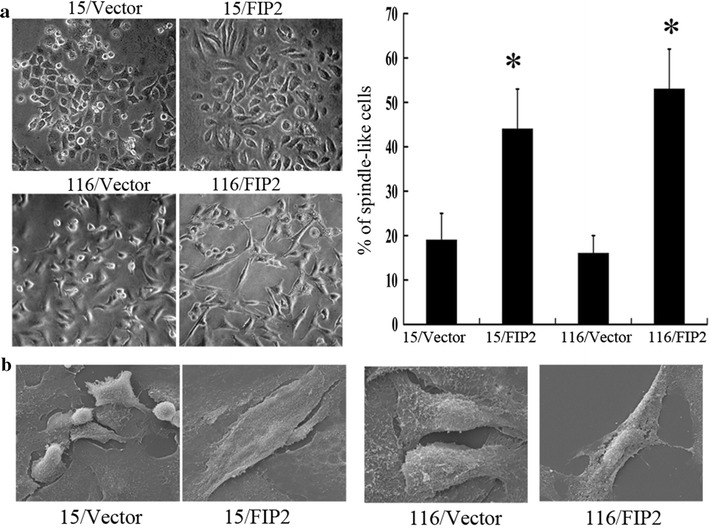
**a** 15/FIP2 and 116/FIP2 cells showed spindle fibroblastic morphology compared with the control cells. The number of spindle-like cells was quantified in three different areas (*p < 0.05). **b** Ectopic expression of Rab11-FIP2 promoted formation of lamellipodia in colorectal cancer (scanning electron microscope: magnification ×3000)

To further validate the tumor promoter function of Rab11-FIP2, we established 15/FIP2 and 116/FIP2 cells which stably overexpressing Rab11-FIP2. The expression of Rab11-FIP2 in 15/FIP2 and 116/FIP2 cells was confirmed by Western Blot (Additional file 1: Figure S1). The effect of Rab11-FIP2 on cell migration was first assessed by wound healing assay. 15/FIP2 and 116/FIP2 cells had significantly faster closure of the wound area compared with their control cells (Fig. 2a). These results were further confirmed by transwell analysis (Fig. 2b). The result of anchorage-independent growth assay revealed that overexpression of Rab11-FIP2 promoted colony formation in CRC cells (Fig. 2c). We also explored if overexpression of Rab11-FIP2 affected metastasis in vivo. The intravenous (i.v.) injection of 15/FIP2 and 116/FIP2 cells developed more nodules in the lungs compared to the control cells (Fig. 2d). Furthermore, lung colonization assays by tail-vein injection of HCT116-Luc cells confirmed overexpression of Rab11-FIP2 promoted lung metastasis in CRC cells (Additional file 2: Figure S2). We also found that 15/FIP2 and 116/FIP2 cells exhibited a spindle-like morphology. Distinct morphologic differences were observed between 15/FIP2 or 116/FIP2 cells and control cells (Fig. 3a). Under a scanning electron microscope, we observed a significant increasement of lamellipodia (cell protrusion) on the cell surfaces of the 15/FIP2 and 116/FIP2 cells compared to respective negative control cells (Fig. 3b).

### Overexpression of Rab11-FIP2 results in increased secretion of PAI-1 in colorectal cells

**Fig. 4:**
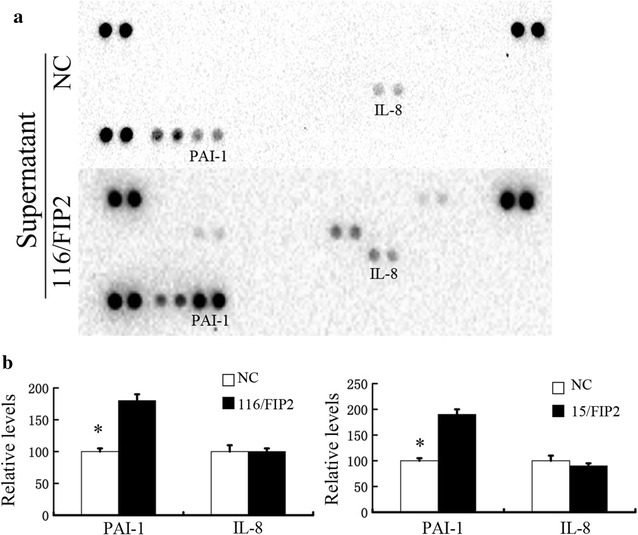
Increased secretion of PAI-1 by overexpression of Rab11-FIP2 in colorectal cells. **a** Antibody array analyses of the supernatant of HCT116 cells expressing a control vector or Rab11-FIP2. Cells were cultured in serum-deprived media (0.1% FBS) for 48 h. The supernatant was collected for detection. Upregulated or downregulated proteins were highlighted. Representative images from two independent experiments were shown. **b** The protein levels of IL-8 and PAI-1 in the supernatant were measured by ELISA. All results are from three independent experiments (*p < 0.05)

**Fig. 5:**
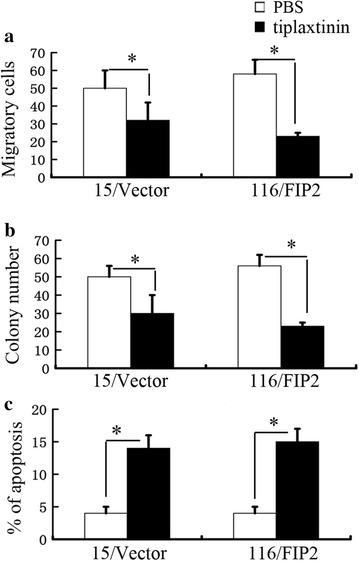
Pharmacological inhibition of PAI-1 resulted in the reduction of cell migration and colony formation, and the induction of apoptosis in CRC overexpressing Rab11-FIP2. **a** Targeting PAI-1 results in a reduction of cell migration in 15/FIP2 and 116/FIP2 cells. **b** Targeting PAI-1 results in a reduction of colony formation in 15/FIP2 and 116/FIP2 cells. **c** Targeting PAI-1 results in an induction of apoptosis in 15/FIP2 and 116/FIP2 cells. All results are from three independent experiments (*p < 0.05)

To further investigate the mechanisms by which Rab11-FIP2 overexpression promotes cell migration, we examined the secreted factors affected by Rab11-FIP2 overexpression using antibody arrays (R&D System, #ARY005B). We cultured 116/FIP2 cells and negative control cells in serum-deprived media (0.1% fetal bovine serum) for 48 h before collecting the cell culture supernatant for antibody array analyses. As shown in Fig. 4a, increased level of PAI-1 was detected in 116/FIP2 cells compared with the negative control cells (Fig. 4a). To confirm these findings, we tested the protein level of PAI-1 in supernatant by using enzyme-linked immunosorbent assay. We detected increased levels of PAI-1 proteins in the conditioned media derived from 15/FIP2 and 116/FIP2 cells compared with respective control cells (Fig. 4b). Additionally, increasing mRNA levels of PAI-1 were found in 15/FIP2 and 116/FIP2 cells but not respective control cells. Our result showed that Rab11-FIP2 up-regulated PAI-1 transcription in CRC (Additional file 3: Figure S3). We also detected the expression levels of Rab11-FIP2 and PAI-1 of 125 CRC specimens. We found that the expression of Rab11-FIP2 was positively correlated with the expression of PAI-1 (r = 0.527, p < 0.001) (Additional file 4: Figure S4). However, the underlying mechanism through which Rab11-FIP2 regulates the expression of PAI-1 at transcription level was totally unknown. PAI-1 has a protumorigenic role in cancer, promoting angiogenesis, tumor invasion, and metastasis [9, 12]. We found that the reduction of cell migration and colony formation, and the induction of apoptosis induced by tiplaxtinin, a PAI-1 inhibitor, were observed in the 15/FIP2 and 116/FIP2 cells (Fig. 5).

### Overexpression of Rab11-FIP2 promotes endothelial cell tube formation

**Fig. 6:**
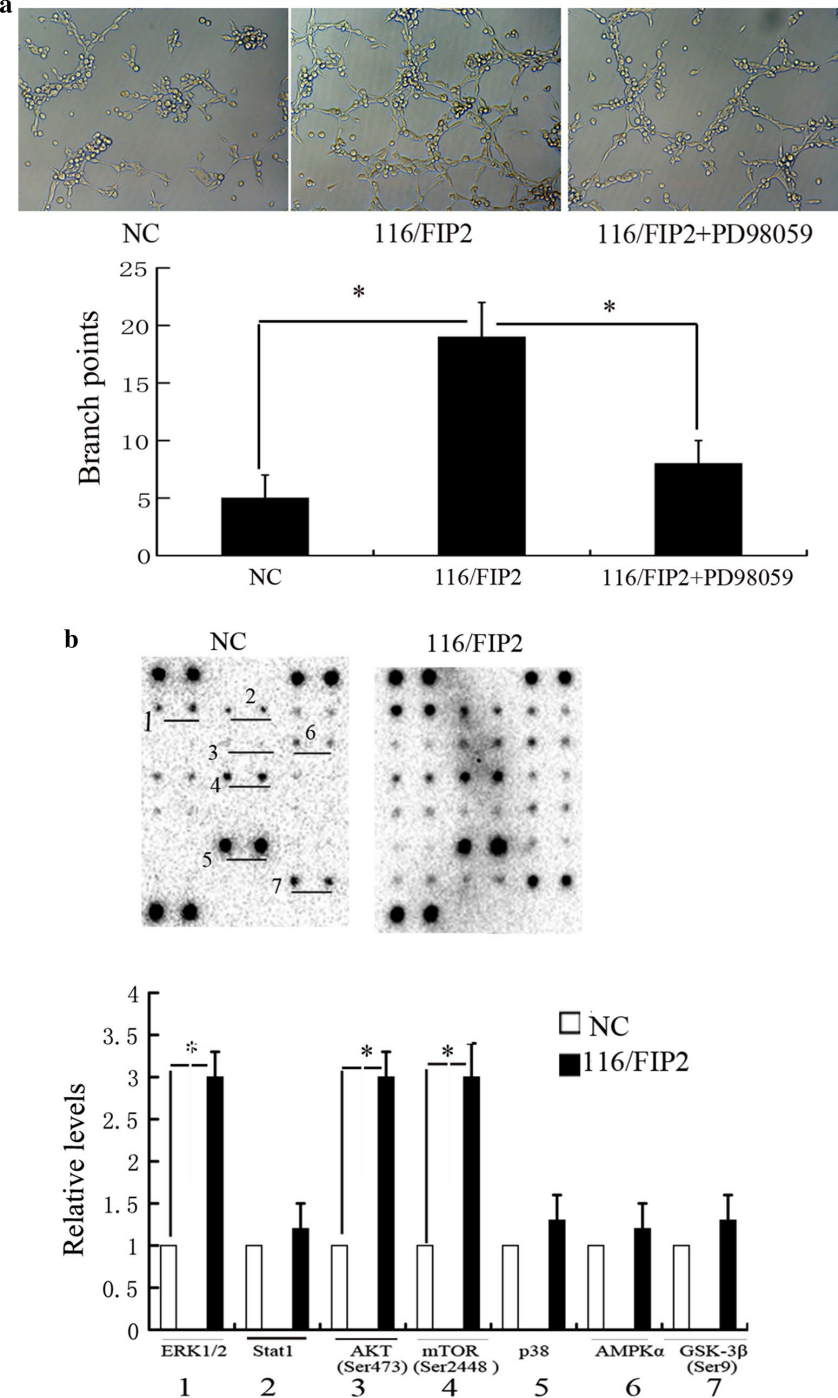
**a** The number of the intersections between branches of assembled endothelial cell networks was observed at ×100 magnification. Branch points numbers were calculated (*p < 0.05). Decreased branch points were observed after PAI-1 inhibition. Data represent the mean of triplicate experiments ± SEM (*p < 0.05). **b** Cells were left untreated or treated with rhPAI-1 for 12 h and lysates applied to anti-phosphotyrosine antibody array. Upregulated proteins were highlighted. Representative images from two independent experiments were shown

To further confirm whether overexpression of Rab11-FIP2 in CRC contribute to angiogenesis, we performed tube-formation assays using human umbilical vein endothelial cells (HUVECs) with matrigel. We observed that conditioned media from HCT116/FIP2 cells induced tube formation of HUVECs. By contrast, conditioned media from control cells failed to support tube formation of HUVECs (Fig. 6a). To test whether overexpressed Rab11-FIP2 contributes to angiogenesis through PAI-1 mediation, we inhibited the function of PAI-1 using tiplaxtinin. Blocking PAI-1 with 50 mmol/L tiplaxtinin inhibited the tube formation induced by conditioned media from HCT116/FIP2 cells. To further confirm that PAI-1 is a critical downstream mediator of Rab11-FIP2-induced endothelial cell tube formation, we also used PAI-1 neutralizing antibody (R&D System, 10 µg/mL) to inhibit the function of PAI-1. In line with the result of tiplaxtinin, blocking PAI-1 with neutralizing antibody inhibited the tube formation induced by conditioned media from HCT116/FIP2 cells (Additional file 5: Figure S5).

**Fig. 7:**
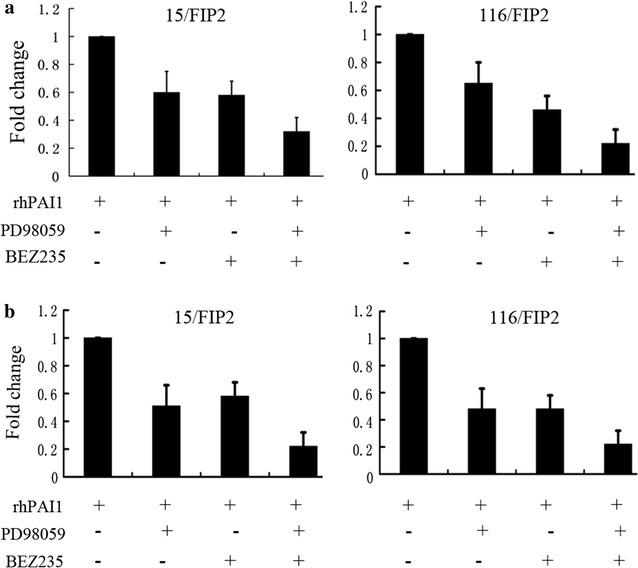
**a** Combinatorial treatment with PD98059 and BEZ235 inhibited migration of CRC more effectively than individual treatment. Data represent the mean of triplicate experiments ± SEM (*p < 0.05). **b** Combinatorial treatment with PD98059 and BEZ235 inhibited migration and clonogenic growth of CRC more effectively than individual treatment. Data represent the mean of triplicate experiments ± SEM (*p < 0.05)

**Fig. 8:**
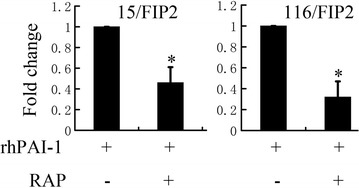
RAP inhibited PAI-1-induced cell migration in 15/FIP2 and 116/FIP2 cells. Data represent the mean of triplicate experiments ± SEM (*p < 0.05)

To further analyze the effect of PAI-1 on CRC with overexpression of Rab11-FIP2, we used an anti-phosphotyrosine antibody array (Cell signaling Technology, #14471) to detect the phosphorylated or cleaved signaling molecules. Our results suggest that the phosphorylation of a group of signaling molecules, including ERK1/2 and AKT/mTOR, is enhanced in 116/FIP2 cells compared with control cells in serum-deprived media (Fig. 6b). We next evaluated the effect of ERK1/2 and AKT/mTOR inhibition alone and in combination on migration and clonogenic growth of CRC. Our results showed that pretreatment with MEK1 inhibitor, PD98059, before rhPAI-1 blocked the PAI-1-induced migration in both 15/FIP2 and 116/FIP2 cells. Pretreatment with mTOR inhibitor, BEZ235 also blocked the PAI-1-induced migration in both 15/FIP2 and 116/FIP2 cells. We also observed a pronounced inhibition on the clonogenic growth following exposure to PD98059 or BEZ235. Furthermore, combinatorial treatment with PD98059 and BEZ235 inhibited migration and clonogenic growth of CRC more effectively than individual treatment (Fig. 7). These results suggest that co-targeting ERK1/2 and AKT/mTOR is more effective in suppressing migration and clonogenic growth of CRC than individual inhibition. PAI-1 binds to the low density lipoprotein receptor-related protein 1 (LRP1) to regulate LRP1-dependent cell motility. For example, LPR1 is essential for PAI-1-mediated FAK phosphorylation and macrophage invasion into melanoma [13]. PAI-1 promoted the migration of microglial cells in culture via the LRP1/JAK/Stat1 axis [14]. We next determined whether LRP1 is involved in the enhancement of cell migration PAI-1. For blocking of LRP1 activity, 15/FIP2 and 116/FIP2 cells were pretreated with two different concentrations of LRP1 antagonist RAP (50 μM) for 30 min prior to migration studies. Although, a small, non-significant decrease was observed in cell migration with the RAP treatment itself, PAI-1-stimulated increase in cell migration was inhibited by the addition RAP (Fig. 8). These results indicate that PAI-1 may increase cell migration in an LRP1-dependent manner.

**Fig. 9:**
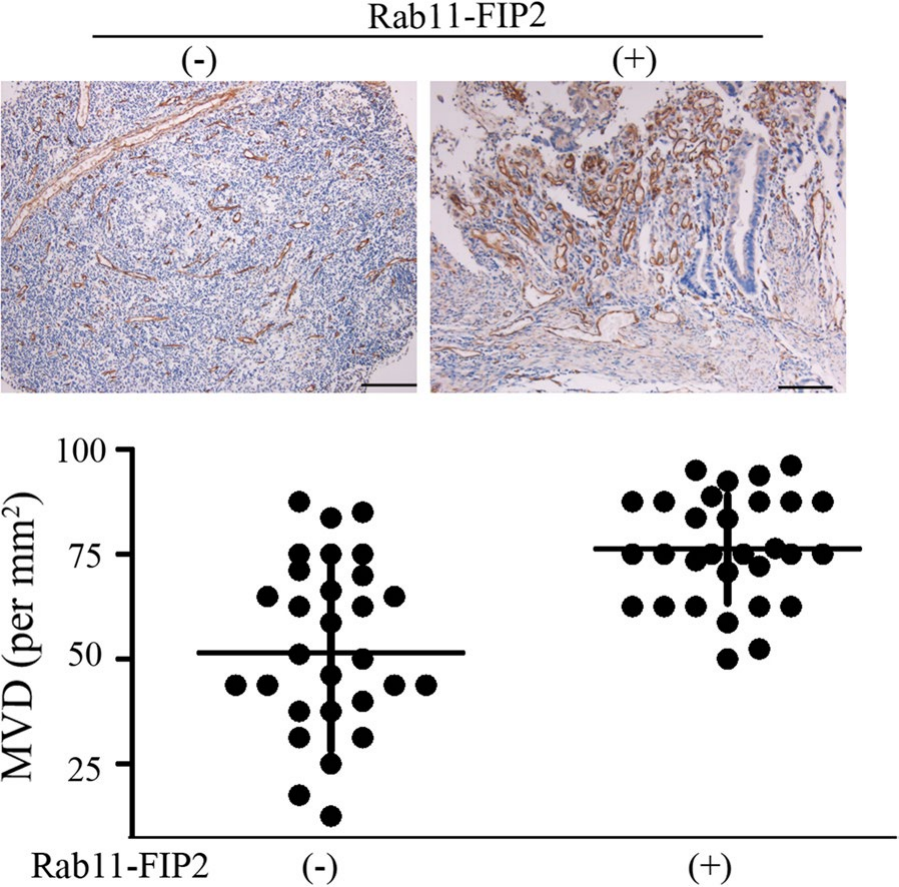
Correlation of Rab11-FIP2 levels with microvessel density (MVD), as determined by quantifying CD34-positive microvessels in colorectal cancer tumor sections. Scale bar = 100 μm. Magnification, ×100 by light microscopy

To evaluate the involvement of Rab11-FIP2 in tumor angiogenesis of CRC clinically, we used immunohistochemical staining for CD34 in 28 human CRC tissue samples and calculated the number of CD34-positive microvessels in the tumor area. We found that the microvessel density (MVD) was positively correlated with the expression of Rab11-FIP2 (Fig. 9). These results imply that Rab11-FIP2 contributes to tumor angiogenesis of CRC.

## Discussion

CRC is the most common malignancy with the third largest incidence and mortality among all diagnosed cancers in the worldwide [15, 16]. In this study, we find that Rab11-FIP2 expression is overexpressed in colorectal cancer and is associated with nodal metastasis. The log rank test demonstrated that tumors with the high Rab11-FIP2 expression were associated with short overall patient survival, whereas patients with tumors displaying low level of Rab11-FIP2 expression showed a better clinical outcome. Metastasis that makes colorectal cancer difficult to treat is the leading cause of cancer mortality. We investigated whether Rab11-FIP2 positively regulates colorectal cancer cells invasion. Our results demonstrated that overexpression of Rab11-FIP2 accelerated cell migration in vitro and tumor metastasis in vivo.

It was reported that Rab11-FIP2 regulated CXCR2 recycling and receptor-mediated chemotaxis, suggesting that Rab11-FIP2 may play a role in chemotaxis [6]. We used an anti-phosphotyrosine receptor antibody array to assess whether RTKs were induced in response to Rab11-FIP2 overexpression. PAI-1, an endogenous inhibitor of urokinase-type plasminogen activator (uPA), is known to play a major role in benign disorders such as deep vein thrombosis, myocardial infarction, atherosclerosis, and stroke, and more recently has been linked to some cancers [17], including colorectal cancer. Perturbation of PAI-1 and the uPA system has been shown to be involved in a number of cancer models, primarily by regulating migration, invasion, apoptosis, and angiogenesis [18, 19]. For example, it was reported that plasma PAI-1 level was increased in CRC patients with liver metastasis, and PAI-1 silencing may suppress colorectal cancer progression and liver metastasis in vitro and in vivo [20]. We found that overexpression of Rab11-FIP2 results in an increased secretion of PAI-1 in colorectal cells. Formation of new blood vessels is crucial for solid tumor growth and metastasis [21]. We found that the microvessel density (MVD) was positively correlated with the expression of Rab11-FIP2. Tumor cells actively release pro-angiogenic factors such as vascular endothelial growth factor to promote endothelial cell proliferation, survival and migration for the formation of new blood vessels [22, 23]. Inhibition of PAI-1 limits tumor angiogenesis regardless of angiogenic stimuli in malignant pleural mesothelioma [9]. Similarly, we found that overexpression of Rab11-FIP2 promotes endothelial cell tube formation in a PAI-1-dependent manner. Collectively, we showed that overexpressed Rab11-FIP2 can induce tumor angiogenesis in CRC, and PAI-1 might mediate this process.

We also found that inhibition of PAI-1 by tiplaxtinin, a specific inhibitor of PAI-1, resulted in the reduction of cell migration and colony formation, and the induction of apoptosis in Rab11-FIP2 overexpression colorectal cancer cells but not the negative control cells. In parallel, we found that there was no significant difference between the PAI-1-treated HCT116 cells with those untreated cells in phosphorylation of signaling molecules (data not shown). It was reported that overexpression of Rab11-FIP2 suppresses the internalization of epidermal growth factor receptors [24]. We postulated that Rab11-FIP2 played an important role in phosphorylation of signaling molecules. Rab11-FIP2 interaction with MYO5B regulates movement of Rab11a-containing recycling vesicles. Rab11-FIP2 has been implicated as a regulator of the recycling of several receptors such as transferrin receptor, the AMPA-type glutamate receptor subunit GluR1, the M4 muscarinic acetylcholine receptor and CXCR2 [5, 25]. PAI-1 binds to the low density lipoprotein receptor-related protein 1 (LRP1) to regulate LRP1-dependent cell motility. We also found that PAI-1 may increase cell migration in an LRP1-dependent manner. We postulated that Rab11-FIP2 activated the phosphorylation of signaling molecules through PAI-1/LRP1 pathway in a vicious cycle manner. Because there were lots works to be done to confirm this postulation, some data was not presented in this article. Importantly, we found that treatment with rhPAI-1 increase the phosphorylation of a group of signaling molecules, including ERK1/2 and AKT/mTOR in 116/FIP2 cells but not the negative control cells, suggesting that overexpression of Rab11-FIP2 increases sensitivity of CRC to PAI-1.

## Conclusion

Our current findings demonstrate a novel role for Rab11-FIP2 in the regulation of colorectal cancer migration. Overexpression of Rab11-FIP2 in colorectal cancer is a strong indicator of aggressive tumors. Uncovering novel functions and the underlying molecular mechanisms of Rab11-FIP2 in colorectal cancer will shed new light on our understanding of tumor metastasis. In conclusion, our findings suggest that Rab11-FIP2 may be a potential target for suppressing colorectal cancer migration under pathologic circumstances. Our results also indicate overexpressed Rab11-FIP2 contributes to tumor angiogenesis through PAI-1 mediation, and combined targeting of Rab11-FIP2 and PAI-1 can control tumor angiogenesis in CRC.

## Additional files

**Additional file 1: Figure S1.** The expression of Rab11-FIP2 in 15/FIP2 and 116/FIP2 cells was confirmed by Western Blot.

**Additional file 2: Figure S2.** Overexpression of Rab11-FIP2 promoted lung metastasis in HCT116-luc cells. Right panel: Representative BLI (Bioluminescence Imaging) images of mice with lung metastasis. HCT116-luc, (colorectal cancer cells expressing luciferase) were preserved in our laboratory and maintained in DMEM with 10% FBS. We established 116/FIP2-Luc cells which stably overexpressing Rab11-FIP2. 116/FIP2-Luc cells and Control cells (1 × 106) were injected into the tail veins of mice. Eight weeks later, the mice were imaged using IVES CCD imaging system. The bioluminescent signal intensity in the region of interest was quantified as total light emission using Living Image Software (Caliper Lifesciences).

**Additional file 3: Figure S3.** RT-PCR analysis was performed for mRNA levels of PAI-1 and GADPH (loading control) in 15/FIP2 and 116/FIP2 cells.

**Additional file 4: Figure S4.** The expression of PAI-1 was also detected by IHC in the tissue samples. We found that the expression of PAI-1 was positively correlated with the expression of Rab11-FIP2 in the tissue samples (r = 0.527, p < 0.001).

**Additional file 5: Figure S5.** HUVECs were seeded on a layer of polymerized Matrigel. Cells were treated with culture media of 116/FIP2 cells with or without PAI-1 neutralizing antibody.

## Abbreviations

Rab11-FIP2: Rab11 family-interacting protein 2
DMEM: Dulbecco’s modified eagle medium
FBS: fetal bovine serum
MVD: microvessel density
