# Supplementary figures and images for: Overexpression of Rab11-FIP2 in colorectal cancer cells promotes tumor migration and angiogenesis through increasing secretion of PAI-1
Source: Cancer Cell Int. 2018 Mar 9;18:35. doi: 10.1186/s12935-018-0532-0 (PMC5845176; doi:10.1186/s12935-018-0532-0)

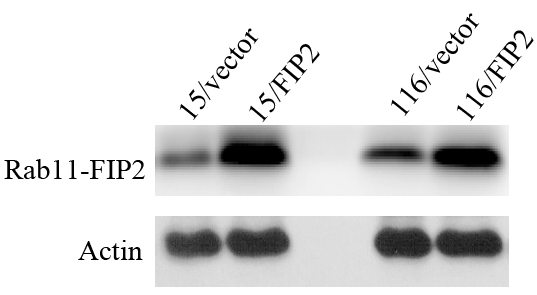

Supplement: Supplementary file 1 — Additional file 1: Figure S1. The expression of Rab11-FIP2 in 15/FIP2 and 116/FIP2 cells was confirmed by Western Blot. [file 12935_2018_532_MOESM1_ESM.jpg]

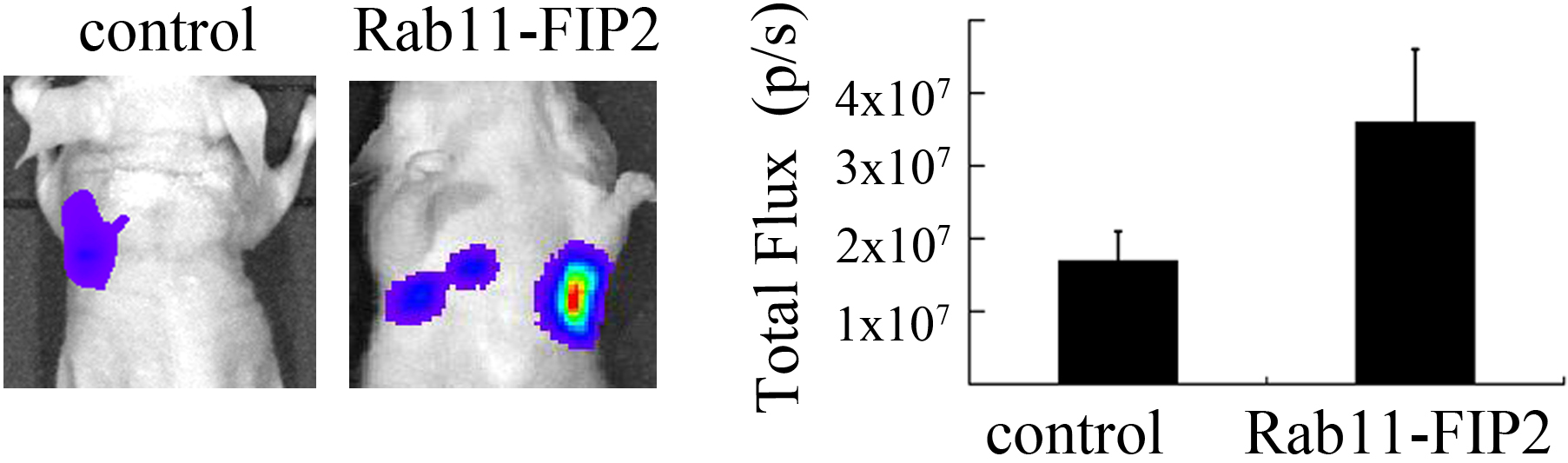

Supplement: Supplementary file 2 — Additional file 2: Figure S2. Overexpression of Rab11-FIP2 promoted lung metastasis in HCT116-luc cells. Right panel: Representative BLI (Bioluminescence Imaging) images of mice with lung metastasis. HCT116-luc, (colorectal cancer cells expressing luciferase) were preserved in our laboratory and maintained in DMEM with 10% FBS. We established 116/FIP2-Luc cells which stably overexpressing Rab11-FIP2. 116/FIP2-Luc cells and Control cells (1 × 10^6^) were injected into the tail veins of mice. Eight weeks later, the mice were imaged using IVES CCD imaging system. The bioluminescent signal intensity in the region of interest was quantified as total light emission using Living Image Software (Caliper Lifesciences). [file 12935_2018_532_MOESM2_ESM.jpg]

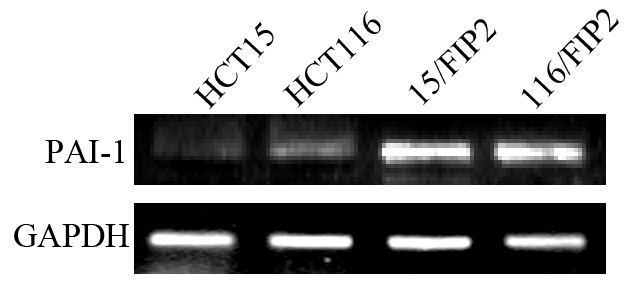

Supplement: Supplementary file 3 — Additional file 3: Figure S3. RT-PCR analysis was performed for mRNA levels of PAI-1 and GADPH (loading control) in 15/FIP2 and 116/FIP2 cells. [file 12935_2018_532_MOESM3_ESM.jpg]

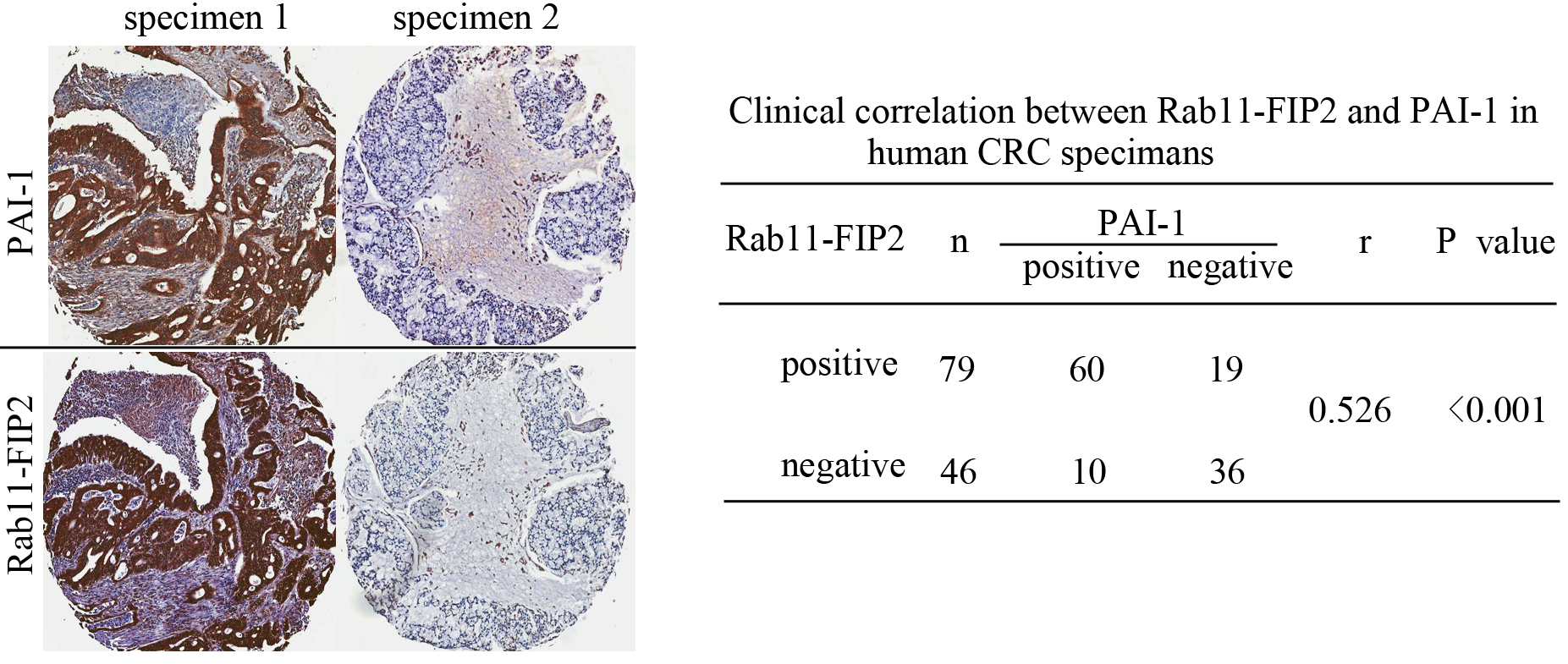

Supplement: Supplementary file 4 — Additional file 4: Figure S4. The expression of PAI-1 was also detected by IHC in the tissue samples. We found that the expression of PAI-1 was positively correlated with the expression of Rab11-FIP2 in the tissue samples (r = 0.527, p < 0.001). [file 12935_2018_532_MOESM4_ESM.jpg]

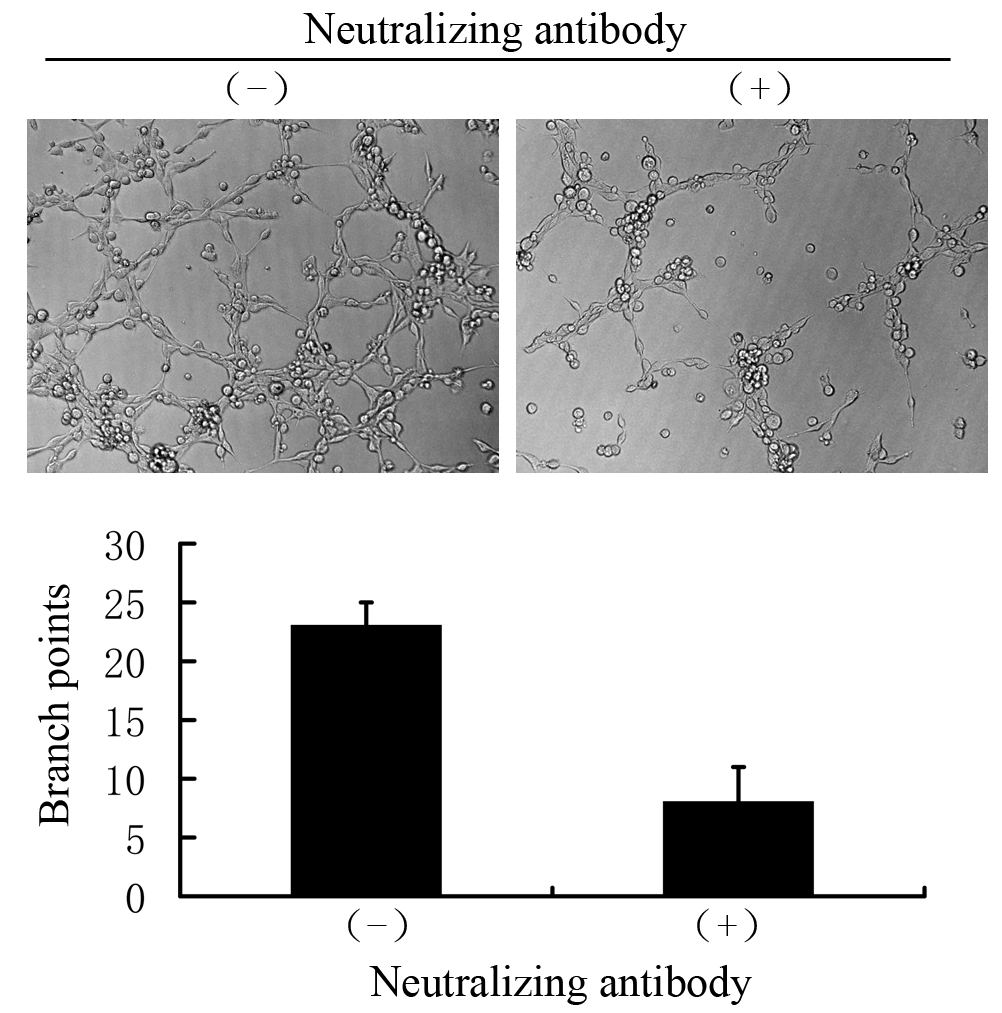

Supplement: Supplementary file 5 — Additional file 5: Figure S5. HUVECs were seeded on a layer of polymerized Matrigel. Cells were treated with culture media of 116/FIP2 cells with or without PAI-1 neutralizing antibody. [file 12935_2018_532_MOESM5_ESM.jpg]
